# Initial surgical management of injuries to the urogenital tract in patients with polytrauma and/or severe injuries: a systematic review and clinical practice guideline update

**DOI:** 10.1007/s00068-025-02847-1

**Published:** 2025-04-29

**Authors:** Christian Ruf, Luis Kluth, Sarah Wahlen, Jessica Breuing, Tim Nestler

**Affiliations:** 1 Department of Urology, Bundeswehr Hospital of Ulm, Ulm, Germany; 2 Department of Urology, University Hospital, Goethe University of Frankfurt, Frankfurt Am Main, Germany; 3 Institute for Research in Operative Medicine (IFOM), Witten/Herdecke University, Cologne, Germany; 4 Department of Urology, Bundeswehr Central Hospital, Rübenacher Str. 170, 56072 Koblenz, Germany; 5 Department of Urology, Faculty of Medicine, Cologne University Hospital, Cologne, Germany

**Keywords:** Surgery, Catheter drainage, Urogenital tract, Genitourinary tract, Polytrauma guideline

## Abstract

**Purpose:**

Our aim was to update evidence-based and consensus-based recommendations for the initial surgical management of urogenital injuries in patients with polytrauma and/or severe injuries based on current evidence. This guideline topic is part of the 2022 update of the German Guideline on the Treatment of Patients with Polytrauma and/or Severe Injuries.

**Methods:**

MEDLINE and Embase were systematically searched to June 2021. Randomised controlled trials, prospective cohort studies, and comparative registry studies were included if they compared surgical and/or therapeutic interventions for urogenital injuries in the hospital setting. We considered patient-relevant clinical outcomes such as mortality and bleeding control, or coagulation parameters as surrogate outcomes. Risk of bias was assessed using NICE 2012 checklists. The evidence was synthesised narratively, and expert consensus was used to develop recommendations and determine their strength.

**Results:**

Two new studies were identified. The topics covered were the comparison of outcomes after surgical and nonsurgical management as well as the use of surgical repair versus catheter drainage in patients with extraperitoneal bladder injuries. Three recommendations were modified, one of which for editorial reasons. All achieved strong consensus.

**Conclusion:**

The following key recommendations are made. 1. Renal artery injuries can be managed using an endovascular approach. 2. Depending on the type and severity of the injury and concomitant injuries, renal injuries should be managed with the intent to preserve the organ. 3. Extraperitoneal bladder ruptures without involvement of the bladder neck should be conservatively treated with catheterisation.

**Supplementary Information:**

The online version contains supplementary material available at 10.1007/s00068-025-02847-1.

## Introduction

In Germany, injuries to the abdomen and pelvis are present in 29.3% of polytraumatised patients with an Abbreviated Injury Scale (AIS) score of at least 2 [1]. A number of abdominal and pelvic injuries can lead to acute life-threatening conditions, which, based on the X-ABCDE priorities of trauma management, affect a patient’s circulation (C). Since especially renal injuries can rapidly result in a life-threatening loss of blood, appropriate treatment in the initial phase of surgical care is of vital importance for patients. A further point of interest is the early surgical management of other types of urological trauma, e.g. bladder or urethral injuries, that are usually not life-threatening but are important for functional outcome. The objective of this systematic review is to identify the current evidence on treatment approaches for urological trauma especially in the initial phase of surgical care and to assess its value as a basis for evidence-based clinical recommendations. This guideline topic is part of the 2022 update of the German Guideline on the Treatment of Patients with Polytrauma and/or Severe Injuries [2].

## Methods

This guideline topic is part of the 2022 update of the German Guideline on the Treatment of Patients with Polytrauma and/or Severe Injuries [3]. The guideline update is reported according to the RIGHT tool [4], the systematic review part according to the Preferred Reporting Items for Systematic Reviews and Meta-Analyses (PRISMA) 2020 reporting guideline [5]. The development and updating of recommendations followed the standard methodology set out in the guideline development handbook issued by the German Association of the Scientific Medical Societies (AWMF) [6]. All methods were defined a priori, following the methods report of the previous guideline version from July 2016 [7] with the modifications detailed below.

### PICO questions and eligibility criteria

Population, intervention, comparison, and outcome (PICO) questions were retained from the previous guideline version. In addition, the participating professional societies involved in guideline development were asked to submit new PICO questions. The overarching PICO question for this topic area was:

*In adolescents / adult patients (*≥*14 years) with known or suspected polytrauma and/or severe injuries and injuries to the genitourinary tract, does a specific initial surgical approach to patient management improve patient-relevant outcomes compared to any other intervention?*

The full set of predefined PICO questions is listed in Table S1 (Online Resource 1). The study selection criteria in the PICO format are shown in Table 1.

**Table 1:** Predefined selection criteria

Population:	*Adolescents/*adult patients (≥ 14 years) with polytrauma and/or severe injuries^a^ and injuries to the genitourinary tract
Intervention/ comparison:	Surgical or interventional measures used in the field of urology
Outcomes:	Any patient-relevant clinical outcomes, such as mortality and bleeding control
Study type:	Comparative, prospective studies (randomised controlled trials, cohort studies) Comparative registry^b^ data (incl. case–control studies) Systematic reviews based on the above primary study types
Language:	English or German
Other inclusion criteria:	Full text of study published and accessible Study matches predefined PICO question
Exclusion criteria:	Multiple publications of the same study without additional information Study already included in previous guideline version

^a^Defined by an Injury Severity Score (ISS) > 15, Glasgow Coma Scale (GCS) < 9, or comparable values on other scales, or, in the prehospital setting, clinical suspicion of polytrauma/severe injury with a need for life-saving interventions

^b^Using the Agency for Healthcare Research and Quality (AHRQ) definition of registries [8]

### Literature search

An information specialist systematically searched for literature in MEDLINE (Ovid) and Embase (Elsevier). The search strategy described in the 2016 guideline update was used with modifications. It contained index (MeSH/Emtree) and free text terms for the population and intervention. Additional terms were included for new PICO questions. The searches were completed on 1 July 2021. The start date was 1 January 2014. Table S2 (Online Resource 1) provides details for all searches. Clinical experts were asked to submit additional relevant references.

### Study selection

Study selection was performed independently by two reviewers in a two-step process using the predefined eligibility criteria: (1) title/abstract screening of all references retrieved from database searches using Rayyan software [9] and (2) full-text screening of all articles deemed potentially relevant by at least one reviewer at the title/abstract level in Endnote (Endnote, Version: 20 [Software], Clarivate, Boston, Massachusetts, USA, https://endnote.com/). Studies limited to the clinical setting were excluded during full-text screening. Disagreements were resolved through consensus or by consulting a third reviewer. The reasons for full-text exclusion were recorded (Table S3, Online Resource 1).

### Assessment of risk of bias and level of evidence

Two reviewers sequentially assessed the risk of bias of included studies at study level using the relevant checklists from the NICE guidelines manual 2012 [10] and assigned each study an initial level of evidence (LoE) using the Oxford Centre for Evidence-based Medicine Levels of Evidence (2009) [11]. For studies with baseline imbalance and unadjusted analyses, post-hoc secondary analyses, indirectness of the study population, or low power and imprecision of the effect estimate, the LoE was downgraded and marked with an arrow (↓). Any disagreements were resolved through consensus or by consulting a third reviewer.

### Data extraction and data items

Data were extracted into a standardised data table by one reviewer and checked by another. A predefined data set was collected for each study, consisting of study characteristics (study type, aims, setting), patient selection criteria and baseline characteristics (age, gender, injury scores), intervention and control group treatments (including important co-interventions), patient flow (number of patients included and analysed), matching/adjusting variables, and data on outcomes for any time point reported.

### Outcome measures

Outcomes were extracted as reported in the study publications. For prospective cohort studies and registry data, preference was given to data obtained after propensity-score matching or statistical adjustment for risk-modulating variables over unadjusted data.

### Synthesis of studies

Studies were grouped by interventions. An interdisciplinary expert group used their clinical experience to synthesise studies narratively by balancing beneficial and adverse effects extracted from the available evidence. Priority was given to reducing mortality, immediate complications, and long-term adverse effects. Clinical heterogeneity was explored by comparing inclusion criteria and patient characteristics at baseline as well as clinical differences in the interventions and co-interventions.

### Development and updating of recommendations

For each PICO question, the following updating options were available: (1) the recommendation of the preceding version remains valid and requires no changes (“confirmed”); (2) the recommendation requires modification (“modified”); (3) the recommendation is no longer valid and is deleted; (4) a new recommendation needs to be developed (“new”). An interdisciplinary expert group of clinicians with expertise in urological trauma, special urological surgery, and reconstructive urology reviewed the body of evidence, drafted recommendations based on the homogeneity of clinical characteristics and outcomes, the balance between benefits and harms as well as their clinical expertise, and proposed grades of recommendation (Table 2). In the absence of eligible evidence, recommendations were made based on clinical experience and expert consensus. These were not graded, and instead labelled as good (clinical) practice points (GPP). For GPPs, the strength of a recommendation is presented in the wording shown in Table 2.

**Table 2:** Grading of recommendations

Symbol	Grade of recommendation	Description	Wording (examples)
⇑⇑	A	Strong recommendation	“use …”, “do not use …”
⇑	B	Recommendation	“should use …”, “should not use …”
⇔	0	Open recommendation	“consider using …”, “… can be considered”

### Consensus process

The Guideline Group finalised the recommendations during a web-based, structured consensus conference on 14 February 2022 via Zoom (Zoom, Version: 5, Zoom Video Communications, Inc., San José, California, USA, https://zoom.us). A neutral moderator facilitated the consensus conference. Voting members of the Guideline Group were delegates of all participating professional organisations, including clinicians, emergency medical services personnel, and nurses, while guideline methodologists attended in a supporting role. Members with a moderate, thematically relevant conflict of interest abstained from voting on recommendations, members with a high, relevant conflict of interest were not permitted to vote or participate in the discussion. Attempts to recruit patient representatives were unsuccessful. A member of the expert group presented recommendations. Following discussion, the Guideline Group refined the wording of the recommendations and modified the grade of recommendation as needed. Agreement with both the wording and the grade of recommendation was assessed by anonymous online voting using the survey function of Zoom. Abstentions were subtracted from the denominator of the agreement rate. Consensus strength was classified as shown in Table 3.

**Table 3:** Classification of consensus strength

Description	Agreement rate
strong consensus	> 95% of participants
consensus	> 75 to 95% of participants
majority approval	> 50 to 75% of participants
no approval	< 50% of participants

Recommendations were accepted if they reached consensus or strong consensus. For consensus recommendations with ≤ 95% agreement, diverging views by members of the Guideline Group were detailed in the background texts. Recommendations with majority approval were returned to the expert group for revision and further discussion at a subsequent consensus conference. Recommendations without approval were considered rejected.

### External review

During a four-week consultation phase, the recommendations and background texts were submitted to all participating professional organisations for review. Comments were collected using a structured review form. The results were then assessed, discussed and incorporated into the text by the guideline coordinator with the relevant author group.

The guideline was adopted by the executive board of the German Trauma Society on 17 January 2023.

### Quality assurance

The guideline recommendations were reviewed for consistency between guideline topic areas by the steering group. Where necessary, changes were made in collaboration with the clinical leads for all topic areas concerned. The final guideline document was checked for errors by the guideline chair and methodologist.

## Results

**Fig. 1:**
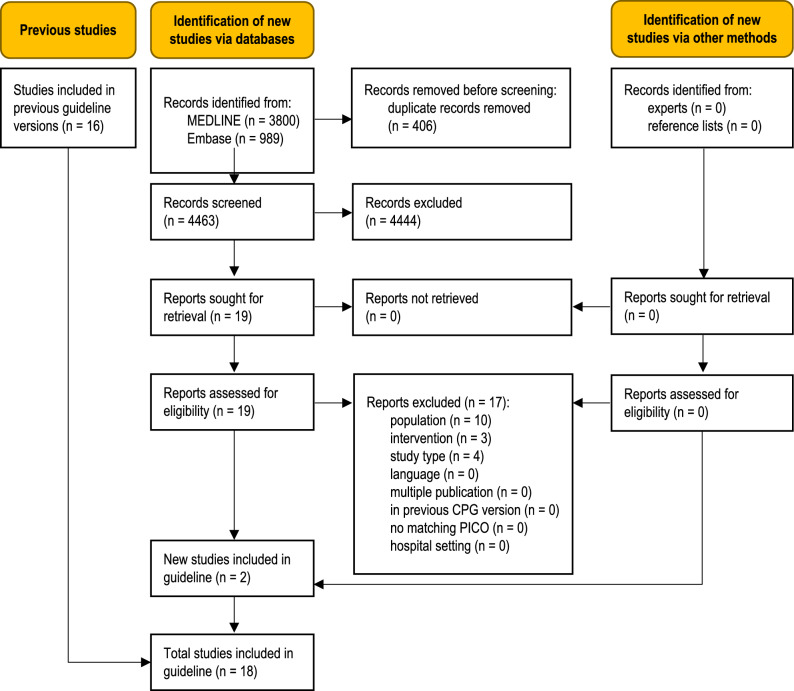
PRISMA 2020 flow diagram showing the systematic literature search and selection of studies

The database searches identified 4789 unique records (Fig. 1). Clinical experts were asked to submit additional references but no relevant publications were provided. Two new studies were eligible for this update [12, 13], adding to the body of evidence from the 16 studies previously included in the guideline [14–29]. A total of 17 full-text articles were excluded (Table S3, Online Resource 1).

### Characteristics of studies included in this update

Study characteristics, main outcomes, levels of evidence, and risk-of-bias assessments are presented in Table 4. Full details are provided in Table S4, Online Resource 1. This update included one comparative registry study [13] and one prospective cohort study [12]. Both studies were performed in North America. Eligible patient populations were adults with severe injuries that involved the urogenital tract [12, 13].

**Table 4:** Characteristics of studies included in the update (see Table S4, Online Resource 1 for details)

Study, ref., design	Population	Interventions (N patients)	Main outcomes (selection)^*^	LoE, risk of bias (RoB)^§^, comments
*Nephrectomy, renal preservation*				
El Hechi 2020 [13] Comparative registry study	Patients with penetrating renal trauma	N = 1842 IG: immediate operation (N = 1512) CG1: NOM (N = 330) CG2: s-NOM (N = 304) CG3: f-NOM (N = 26)	*Severe sepsis* *n (%)* IG: 71 (4.7) CG1: 1 (0.3), p < 0.001 CG2: 1 (0.3) CG3: 0 (0.0), p = 1.00 IG: 71 (4.7) CG2: 1 (0.3), p = n.r *Hospital days [d], median (IQR)* IG: 14.0 (8.0, 25.0) CG1: 6.0 (4.0, 12.0), p < 0.001 CG2: 6.0 (4.0, 10.0) CG3: 20.0 (11.0, 34.0), p < 0.001 IG: 14.0 (8.0, 25.0) CG2: 6.0 (4.0, 10.0), p = n.r *Need for dialysis* *n (%)* IG: 42 (2.8) CG1: 0 (0.0), p < 0.001 CG2: 0 (0.0) CG3: 0 (0.0), p = n.a IG: 42 (2.8) CG2: 0 (0.0), p = n.r *Inpatient morbidity* *n (%)* IG: 482 (31.9) CG1: 37 (11.2), p < 0.001 CG2: 27 (8.9) CG3: 10 (38.5), p < 0.001 IG: 482 (31.9) CG2: 27 (8.9), p = n.r	LoE 2b High risk of bias
*Management of extraperitoneal bladder ruptures*				
Anderson 2020 [12] Prospective cohort study	Patients with extraperitoneal bladder injuries	N = 157 IG: catheter drainage (N = 90) CG: operative repair (N = 67)	*Length of stay [d], median (IQR)* IG: 12 (6–20) CG: 12 (6–21), p = 0.81 *ICU length of stay [d], median (IQR)* IG: 5 (3–13) CG: 6 (2–15), p = 0.97 Urologic complications n (%)** IG: 16 (18) CG: 11 (16), p = 0.82	LoE 2b High risk of bias

*Data for IG versus CG unless otherwise specified. ^§^ Risk of bias: low RoB = RoB low for all domains; unclear RoB = RoB unclear for at least one domain, no high RoB in any domain; for studies with high RoB, all domains with high RoB are named, with RoB low or unclear for all other domains (for full details Table S4, Online Resource 1). For abbreviations and acronyms see list included

**Significant complications were defined as the presence of the following urologic or orthopaedic conditions: pelvic infection/urinoma, persistent urinary extravasation, urinary tract fistula, non-union fractures, hardware infection or removal, and pelvic osteomyelitis

### Risk-of-bias assessment for included studies and levels of evidence

The risk of bias was high for both studies owing to a lack of details regarding selection and performance bias. Both studies had an unclear risk of detection bias.

### Recommendations

Three recommendations were modified, one of which for editorial reasons only (Table 5). All achieved strong consensus.

**Table 5:** List of recommendations with grade of recommendation and strength of consensus

No	GoR	New evidence, consensus^a^	Recommendation	Status 2022
*Urogenital tract*				
1	B ⇑	– 100%	AAST grade V renal injuries, which represent the most severe renal injuries, should be surgically explored	Confirmed
2	B ⇑	– 100%	In haemodynamically stable patients, renal injuries (< grade V) should initially be managed conservatively	Confirmed
3	0 ⇔	– 100%	Intermediate-grade renal injuries (grade III or IV) can be surgically explored in patients who require laparotomy for other injuries	Confirmed
4	0 ⇔	– 100%	Renal artery injuries can be managed using an endovascular approach	Modified
5	B ⇑	[13] 100%	Depending on the type and severity of the injury and concomitant injuries, renal injuries should be managed with the intent to preserve the organ	Modified
6	B ⇑	– 100%	Primary nephrectomy should be performed for grade V injuries only	Confirmed
*Bladder injuries*				
7	B ⇑	– 100%	Intraperitoneal bladder ruptures should be surgically explored	Confirmed
8	B ⇑	[12] 100%	Extraperitoneal bladder ruptures without involvement of the bladder neck should be conservatively treated with catheterisation	Modified
*Urethral injuries*				
9	B ⇑	– 100%	Complete urethral ruptures should be managed with suprapubic catheterisation in the initial phase of surgical care	Confirmed
10	0 ⇔	– 100%	Urethral realignment can be performed in addition to suprapubic catheterisation for urinary drainage	Confirmed
11	B ⇑	– 100%	If surgery is required for a pelvic fracture or another intra-abdominal injury, a urethral rupture should be treated in the same session	Confirmed

*AAST* American Association for the Surgery of Trauma, *GoR* grade of recommendation

^a^Recommendations 1–3: consensus of 16 voting members of the Guideline Group; recommendations 4–11: consensus of 18 voting members

## Discussion

### Rationale for recommendations

In the following sections, the current recommendations regarding the initial surgical management of renal, ureteral, bladder and urethral injuries are discussed.

### Renal injuries

Surgical exploration of renal trauma is required for haemodynamical instability and blood loss necessitating transfusion and is also determined by serum creatinine levels and injury severity [30]. In addition, the decision to perform surgical exploration depends on whether other abdominal injuries require observation or exploration [31].

Haemodynamical instability is an absolute indication for exploration [32, 33]. Further indications are expanding or pulsatile perirenal haematomas greater than 3.5 cm, contrast extravasation, and grade IV or V renal trauma (see Table 5, recommendations 1–3) [32, 33]. The first and foremost objective of surgical exposure of renal trauma is bleeding control and then, if possible, suturing of the defect (renorrhaphy) and perirenal drainage of a haematoma or urinoma. In the majority of cases, exploration in haemodynamically unstable patients results in nephrectomy [34, 35]. One of the tools informing the decision to perform surgical exploration is the revised classification of renal injuries according to Moore et al. and Buckley and McAninch [36, 37]. Surgical exploration is currently performed in approximately 10–15% of cases. This rate would decrease further if more centres advocated nonoperative treatment approaches for renal trauma [38, 39]. A transperitoneal approach is most commonly recommended for surgical exploration [40, 41]. In these cases, access to the renal pedicle is obtained through the posterior parietal peritoneum, which is incised medial to the inferior mesenteric vein [41]. Temporary clamping of the renal pedicle prior to opening Gerota’s fascia is a safe and effective procedure for the exploration and reconstruction of renal trauma [42, 43]. Temporary clamping lowers intraoperative blood loss and the rate of nephrectomy [41].

In the past decade, advances in haemorrhage control interventions have decisively influenced the management of renal trauma. Angiography with selective embolisation has become the most important alternative option to surgical exploration if there are no other indications for laparotomy (see Table 5, recommendation 4) [33]. In the most recent European Association of Urology (EAU) guidelines, angiography with selective embolisation is recommended as the first-line treatment [33]. Previously, the use of angiography was limited to the management of secondary or isolated trauma as documented in a few case series and case reports [44, 45]. It is undisputed that patient selection, technical equipment and the surgeon’s personal experience have a decisive influence on the rate of success. The decision to opt for an endovascular procedure is based on the AAST classification of renal injuries, which depends on the results of computed tomography (CT). Patients with multiple injuries undergo whole-body CT.

Technical improvements in angiography equipment, catheter and embolisation material have led to better outcomes and the increasing use of endovascular therapies also for grade IV and V renal injuries after blunt trauma [46]. In a recent systematic review that was conducted in accordance with PRISMA guidelines and included 16 retrospective studies and a total of 412 patients with renal injuries of AAST grade II (2%), grade III (23%), grade IV (55%), or grade V (20%), endovascular therapy (angioembolisation) was successful in 92% of all grade III and IV injuries and in 76% of all grade V injuries [47]. In addition, it had a success rate of 90% in haemodynamically stable patients but only 63% in haemodynamically unstable patients. For this reason, the authors agree with other researchers who recommend that haemodynamically stable patients with grade III to V renal injuries undergo endovascular therapy (angiography and selective embolisation) [44, 45, 47, 48].

During the past decades, endovascular therapy has played an increasingly important role in the management of blunt renal injuries. This, however, does not apply to penetrating renal injuries. Endovascular procedures are rarely used for penetrating renal injuries.

Most groups of researchers agree that the following findings of mandatory CT are indications for angiography with selective embolisation [33, 47-49]:

- active contrast extravasation from a main renal artery, a segmental or subsegmental renal artery,
- large perirenal haematoma,
- AAST grade III, IV and V renal trauma,
- interruption of Gerota’s fascia,
- arteriovenous fistulas or aneurysms.

Although injuries to the renal pedicle often require a surgical intervention, endovascular treatment options play an increasingly important role in the management of these injuries. In cases of severe multiple trauma or high operative risk, for example, a main renal artery may be embolised in order to achieve proximal occlusion as a definitive (damage control) treatment or as a procedure that is followed by interval nephrectomy after stabilisation of the patient [33].

In haemodynamically stable patients, parenchymal reconstruction is usually possible (see Table 5, recommendation 5) [50]. Nephrectomy is mainly required in patients with penetrating injuries, high transfusion requirements, haemodynamical instability, and high Injury Severity Scores [51]. In general, mortality is more strongly associated with overall trauma severity than with the renal injury itself [33].Reconstruction is difficult in injuries caused by high-velocity projectiles. These injuries usually require nephrectomy [33]. If reconstruction is possible, renorrhaphy is the most common reconstructive technique [41]. Nonviable parenchymal tissue may require secondary partial nephrectomy [33].

In 2020, El Hechi et al. analysed data from the American College of Surgeons Trauma Quality Improvement Program (ACS-TQIP) and found significant differences between the operative and the nonoperative management of penetrating renal trauma in terms of sepsis, hospital length of stay, intensive care unit (ICU) length of stay, duration of ventilation, inpatient morbidity, need for dialysis, acute kidney injury, and ventilator-associated pneumonia. The organ-preserving approach was found to be highly successful. Immediate nephrectomy was superior to nonoperative treatment in the management of grade IV and V injuries [13]. Based on these data, the guideline recommendation that, depending on injury type and severity, renal injuries be managed with the intent to preserve the organ was upgraded from Grade 0 (“can”) to Grade B (“should”) (see Table 5, recommendation 5).

Injuries to the renal pedicle are usually associated with extensive trauma and elevated morbidity and mortality rates [33]. Only in extremely rare cases is it technically possible to reconstruct grade V injuries of the renal pedicle. Repair, however, should be attempted in patients with a solitary kidney or bilateral renal injuries (see Table 5, recommendation 6) [33]. In general, patients with grade V injuries to the renal pedicle should be treated with primary nephrectomy [33].

### Ureteral injuries

Ureteral injuries from trauma are uncommon. In the absence of sufficient evidence, the current guideline does not provide recommendations on the management of these injuries. Depending on injury severity, ureteral injuries are divided into five grades (see Table 6: Classification of Ureteral Injury According to AAST).

**Table 6:** Classification of Ureteral Injury According to AAST

Grade I: Hematoma without devascularization
Grade II: Laceration with less than 50% transection
Grade III: Laceration with more than 50% transection
Grade IV: Laceration with complete transection and less than 2 cm devascularization
Grade V: Laceration or avulsion with more than 2 cm devascularization

Partial ureteral injuries can be defined as grade I and II injuries [52]. Following initial diagnosis, low grade injuries can be managed with ureteral stenting or percutaneous nephrostomy tube placement [53]. Ureteral stents, which can be placed both in an antegrade and a retrograde fashion, stabilise the defect and prevent the formation of strictures. They should be left in place for three weeks [53, 54]. Especially if there is a delay in diagnosis, percutaneous nephrostomy is recommended as a primary procedure. It allows an antegrade stent to be placed either at the time of nephrostomy or after two to seven days [54].

Retrospective comparative studies show that an antegrade approach has a higher chance of success and is easier to perform [53]. This applies in particular to higher-grade injuries (grade II and III) [53]. Following successful stent placement, an open surgical procedure is required only for persistent extravasation or strictures [53].

If grade II or III injuries are detected during open exploration, they can be managed with primary suturing after stent placement [53]. Gunshot or stab wounds, however small they may be, should never be repaired with primary suturing, since safe debridement is essential for complete healing without stricture formation [53].

### Bladder injuries

In the majority of cases, the many concomitant injuries that are frequently seen in multiply injured patients must be managed before bladder injuries are addressed. Extraperitoneal injuries occur twice as often as intraperitoneal bladder ruptures [20, 55]. Combined extraperitoneal and intraperitoneal ruptures are far less common [56]. Combined extraperitoneal and intraperitoneal ruptures are far less common [47]. In general, treatment approaches distinguish between blunt and penetrating as well as intraperitoneal and extraperitoneal bladder ruptures. According to the AAST, bladder injuries are classified into five grades (see Table 7: Classification of Bladder Injury According to AAST).

**Table 7:** Classification of Bladder Injury According to AAST

Grade I: Hematoma or partial laceration of the bladder wall
Grade II: Extraperitoneal tear of the bladder wall less than 2 cm
Grade III: Extraperitoneal tear of the bladder wall greater than 2 cm, or intraperitoneal tear of the bladder wall less than 2 cm
Grade IV: Intraperitoneal tear of the bladder wall greater than 2 cm
Grade V: Bladder injury extending into the bladder trigone or bladder neck/sphincter region

#### Blunt trauma

##### Extraperitoneal bladder injuries

Uncomplicated bladder injuries (grade I to III) can usually be managed using a transurethral catheter, regardless of the presence or absence of extensive perineal or scrotal extravasation [57, 58]. Exceptions include bladder neck injuries, bone fragments in the bladder wall, entrapment of the bladder wall, or concomitant rectal injuries (grade V) that should be managed surgically [57, 58]. If a surgical exploration is conducted for other injuries, extraperitoneal bladder ruptures should also be treated operatively with a view to minimising the risk of infection [59, 60].

In a prospective cohort study from 2020, Anderson et al. investigated initial management strategies for extraperitoneal bladder injuries. They compared operative repair and catheter drainage in terms of hospital length of stay, ICU length of stay, and urologic and orthopaedic complications. They found no significant differences between suprapubic cystostomy and surgery [12]. If suprapubic cystostomy is not possible, transurethral catheter drainage can be performed too. As a result of this study, the guideline recommendation that extraperitoneal bladder ruptures without involvement of the bladder neck be conservatively treated through urinary diversion was upgraded from Grade 0 (“can”) to Grade B (“should”) (see Table 5, recommendation 8).

#### Intraperitoneal bladder injuries

Intraperitoneal bladder injuries (grade III and V) should always be managed by primary surgical repair because of the risk of peritonitis or sepsis and the associated high mortality (see Table 5, recommendation 7) [57, 58, 60].

##### Penetrating trauma

Penetrating bladder injuries require immediate surgical exploration [58, 61]. Midline cystostomy is the recommended approach. It allows the bladder wall to be inspected and the bladder neck and distal ureters to be examined for concomitant injuries [57, 61].

#### Extensive bladder injuries

Bladder reconstruction using a myocutaneous flap is an option for the management of extensive bladder wall defects, for example resulting from avulsion of the lower abdominal wall or the perineum with involvement of the bladder wall [57, 62].

##### General intraoperative and postoperative management

If possible, bladder injuries should be surgically repaired with a two-layer mucosa-detrusor suture using absorbable material [57, 63]. Postoperative bladder drainage lowers intravesical pressure and aids in the tension-free approximation of the wound edges [63]. Depending on the type and extent of injury, a urinary catheter should be left in place for seven to fourteen days [57, 63].

If possible, retrograde cystography should be performed prior to catheter removal [57, 63]. If contrast extravasation is detected, the catheter can remain in place for a further seven days and cystography should then be repeated [57, 63].

### Urethral injuries

It should be noted that this section explicitly addresses the management of urethral injuries during the initial phase of surgical care. Different principles apply in later phases of care.

Currently there is no evidence that sufficiently demonstrates that primary, delayed or secondary re-anastomosis is preferable in the management of complete posterior urethral ruptures. Chapple et al. proposed deferred urethral repair [64]. The main post-traumatic problems are urethral strictures, incontinence and impotence. The objective of treatment is to prevent these complications.

Koraitim conducted a literature review that included several case series and comparative studies addressing different treatments for urethral ruptures. He reported that suprapubic cystostomy alone was associated with a stricture rate of 97%, an incontinence rate of 4%, and an impotence rate of 19%. By contrast, primary alignment was associated with rates of 53%, 5% and 36%, respectively, and primary suturing with rates of 49%, 21% and 56%, respectively [31, 65–71]. On the basis of these data, it is recommended that complete urethral ruptures in male patients be managed with suprapubic catheterisation alone or with realignment if there is a marked separation of the urethral ends (see Table 5, recommendations 9 and 10) [69]. In a more recent study, Ku et al. too found that both treatments were equally effective [72].

If surgery is required for injuries adjacent to the urethra, it can be appropriate to treat a urethral rupture in the same session. A two-stage procedure can thus be avoided (see Table 5, recommendation 11) [73]. Especially if colonic injuries lead to contamination of the abdominal cavity, primary suturing of the urethra over a stenting catheter may be effective in order to avoid complicating infections [74]. Even if conservative management appears possible, urethral injuries can be treated with primary surgery if definitive fixation of the bony pelvis cannot otherwise be performed [75].

In males, anterior urethral ruptures are less common than posterior urethral ruptures [74]. Open injuries may require primary surgical reconstruction. In most cases, however, suprapubic cystostomy and delayed repair are preferable since the reconstruction of the anterior urethra and the male external genitalia, which are also often affected, is usually difficult and time-consuming (see Table 5, recommendation 9) [64]. In cases of penile fractures associated with injuries to the corpora cavernosa, however, it is recommended that urethral injuries be treated with primary surgery too [64, 76]. The decision whether to perform primary surgery or to use a conservative approach is guided by the severity of the urological injury and overall injury severity [64, 77].

Urethral injuries are considerably less common in females than in males. When these injuries occur, however, they are usually severe and associated with bladder injuries [67]. For this reason, initial treatment consists of suprapubic catheterisation alone if the patient is haemodynamically unstable and/or other injuries require more urgent surgical treatment [78]. By contrast, proximal urethral ruptures in female patients with less severe multiple trauma can be managed with primary reconstruction using a retropubic approach [79–81].

These recommendations similarly apply to children. Again, gender differences should be considered. In 1997, Podestá et al. compared suprapubic cystostomy and delayed urethroplasty, suprapubic cystostomy and urethral realignment, and primary anastomosis in a series of 35 boys with posterior urethral disruption [82]. Since primary anastomosis was associated with a continence rate of only 50% and all ten patients who underwent urethral realignment required delayed urethroplasty, the authors recommended suprapubic cystostomy as the only initial treatment followed by delayed urethroplasty [29]. These authors also conducted a study on urethral injuries in girls with pelvic fractures and other concomitant injuries and found that delayed surgical treatment resulted in good outcomes despite of concomitant bladder and vaginal injuries [83].

### Limitations of the guideline

Patient values and preferences were sought but not received. The effect of this on the guideline is unclear, and there is a lack of research evidence on the effect of patient participation on treatment decisions or outcomes in the emergency setting.

### Unanswered questions and future research

In centres with the necessary expertise, nonoperative interventional radiological techniques are used today for the management of grade V renal trauma in selected clinically stable patients. There are, however, no studies that provide sufficient evidence to recommend this approach. Moreover, there is no evidence that sufficiently demonstrates that primary, delayed or secondary re-anastomosis is preferable in the management of complete posterior urethral ruptures.

## Supplementary Information

Below is the link to the electronic supplementary material.

Supplementary file1 (DOCX 25 KB)

## Data Availability

No datasets were generated or analysed during the current study.

## Abbreviations

AAST: American Association for the Surgery of Trauma
ACS-TQIP: American College of Surgeons Trauma Quality Improvement Program
AHRQ: Agency for Healthcare Research and Quality
CG: Control group
d: Days
EAST: Eastern Association for the Surgery of Trauma
EAU: European Association of Urology
f-NOM: Failure of nonoperative management
GoR: Grade of recommendation
GCS: Glasgow Coma Scale
ICU: Intensive care unit
IG: Intervention group
IQR: Interquartile range
LoE: Level of evidence
n.a.: Not applicable
NOM: Nonoperative management
n.r.: Not reported
PRISMA: Preferred Reporting Items for Systematic Reviews and Meta-Analyses
SD: Standard deviation
s-NOM: Successful nonoperative management
y: Years
