# Supplementary material for: Initial surgical management of injuries to the urogenital tract in patients with polytrauma and/or severe injuries: a systematic review and clinical practice guideline update
Source: Eur J Trauma Emerg Surg. 2025 Apr 29;51(1):182. doi: 10.1007/s00068-025-02847-1 (PMC12037646; doi:10.1007/s00068-025-02847-1)
Supplement: Supplementary file 1 — Supplementary file1 (DOCX 25 KB) [file 68_2025_2847_MOESM1_ESM.docx]

3.6 Urogenital tract

# S1 PICO Questions

|  | Population | Intervention(s) | Control(s) | Outcome(s) |
| --- | --- | --- | --- | --- |
| 1 | Target population, with kidney injury grade V according to AAST | Surgical exploration | Conservative | Patient-relevant outcomes |
| 2 | Target population, (circulatory stable) with kidney injury <Grade V after AAST. | Conservative | Operative | Patient-relevant outcomes |
| 3 | Target population, with kidney injury grade III or IV according to AAST and indication for laparotomy | Surgical exploration | Conservative | Patient-relevant outcomes |
| 4 | Target population, (circulatory stable) with renal arterial injury | Therapeutic, selective angiographic embolisation | Conservative, other procedure | Patient-relevant outcomes |
| 5 | Target population, with renal injury | Suturing, partial kidney resection, organ-preserving surgery | Other procedure | Patient-relevant outcomes |
| 6 | Target population, with grade V renal injury | Primary nephrectomy | Other procedure, suturing, partial kidney resection, organ-preserving surgery | Patient-relevant outcomes |
| 7 | Target population, with intraperitoneal urinary bladder rupture | Surgical exploration | Suprapubic urinary diversion | Patient-relevant outcomes |
| 8 | Target population, with extraperitoneal urinary bladder ruptures without involvement of the bladder neck | Suprapubic urinary diversion | Surgical procedure/exploration | Patient-relevant outcomes |
| 9 | Target population, with urethral ruptures | Suprapubic urinary diversion (with urethral splinting if necessary) | Other procedure | Patient-relevant outcomes |
| 10 | Target population, with urethral ruptures with pelvic or other intra-abdominal injuries | Urethral curettage in one surgical session | Subsequent treatment/surgery of urethral rupture | Patient-relevant outcomes |

# S2 Literature Search

| Suchstrategie 2021, MEDLINE (via Ovid) Datum: 01.07.2021 3.880 Treffer |
| --- |
| 1. *kidney/in, su or *urethra/in, su or *urinary bladder/in, su or (((urethra* or bladder or kidney or renal) adj2 (injur* or trauma*)) or ((urethra* or bladder) adj1 rupture)).ti,ab,kf.  2. exp Multiple Trauma/  3. (polytrauma* or trauma patient?).ti,ab,kf. or (severe adj2 shock).ti,ab,kf.  4. ((multiple or major or severe* or serious*) adj3 (trauma* or injur*)).ti,ab,kf.  5. ((blunt or penetrating) adj5 (trauma* or injur*)).ti,ab,kf.  6. (*Critical Care/ or *Emergencies/ or (life threatening or critical* care or emergen*).ti,ab,kf.) and (trauma* or injur*).ti,ab,kf.  7. 2 or 3 or 4 or 5 or 6  8. 1 and 7  9. exp animals/ not humans.sh.  10. 8 not 9  11. (comment or editorial or letter).pt. or case report*.mp.  12. 10 not 11  13. limit 12 to dt=20090101-20210731 |
| Suchstrategie 2021, Embase (via Elsevier) Datum: 01.07.2021 989 Treffer |
| #1 'kidney injury'/exp OR 'urethra injury'/exp OR 'bladder'/exp OR (((urethra* OR bladder OR kidney OR renal) NEAR/2 (injur* OR trauma*)) OR ((urethra* OR bladder) NEXT/1 rupture)):ti,ab,kw  #2 'multiple trauma'/exp  #3 (polytrauma* OR "trauma patient?"):ti,ab,kw OR (severe NEXT/2 shock):ti,ab,kw  #4 ((multiple OR major OR severe* OR serious*) NEXT/3 (trauma* OR injur*)):ti,ab,kw  #5 ((blunt OR penetrating) NEXT/5 (trauma* OR injur*)):ti,ab,kw  #6 ('intensive care'/mj OR 'emergency'/mj OR ("life threatening" OR "critical care" OR emergen*):ti,ab,kw) AND (trauma* OR injur*):ti,ab,kw  #7 #2 OR #3 OR #4 OR #5 OR #6  #8 #1 AND #7  #9 'animals'/exp NOT 'humans'/de  #10 #8 NOT #9  #11 (comment OR editorial OR letter):it OR "case report*":ti,ab,kw  #12 #10 NOT #11  #13 [1-1-2009]/sd NOT [1-8-2021]/sd  #14 #12 AND #13  #15 [embase]/lim  #16 #14 AND #15  #17 embase NOT (embase AND medline)  #18 #16 AND #17  #19 #18 AND ('article'/it OR 'article in press'/it OR 'erratum'/it OR 'review'/it) |

# S3 Excluded Studies

| **Reference** | **Reason for exclusion** |
| --- | --- |
| Anderson RE, Keihani S, Das R, Hanson HA, McCrum ML, Hotaling JM, et al. Nephrectomy is Associated with Increased Mortality after Renal Trauma: An Analysis of the National Trauma Data Bank from 2007-2016. Journal of Urology. 2021;205(3):841-7. | Study population: adult patients (≥ 14 years) with polytrauma or trauma-related severe injury. |
| Barrett K, Braga LH, Farrokhyar F, Davies TO. Primary realignment vs suprapubic cystostomy for the management of pelvic fracture-associated urethral injuries: a systematic review and meta-analysis. Urology. 2014;83(4):924-9. | Study type: comparative, prospective studies, comparative registry data, case-control studies (no non-comparative-studies and no retrospective cohort studies) & cross-sectional studies as well as Systematic Reviews* (based on the aforementioned primary stentypen), die relevante (klinische) Endpunkte berichten. |
| Bjurlin MA, Fantus RJ, Fantus RJ, Villines D. Comparison of nonoperative and surgical management of renal trauma: Can we predict when nonoperative management fails? The Journal of Trauma and Acute Care Surgery. 2017;82(2):356-61. | Study population: adult patients (≥ 14 years) with polytrauma or trauma-related severe injury. |
| Chavez-Iniguez JS, Ibarra-Estrada MA, Claure-Del Granado R, de Quevedo AA, Maggiani-Aguilera P, Cervantes-Sanchez C, et al. Acute kidney injury in renal trauma patients. Clinical Nephrology. 2021;95(3):143-50. | Intervention: surgical/interventional measures in the field of urology. |
| Deibert CM, Spencer BA. The association between operative repair of bladder injury and improved survival: results from the National Trauma Data Bank. Journal of Urology. 2011;186(1):151-5. | Study population: adult patients (≥ 14 years) with polytrauma or trauma-related severe injury. |
| Gor RA, Styskel BA, Li T, Canter DJ, Simhan J. Unexpected High Rates of Angiography and Angioembolization for Isolated Low-grade Renal Trauma: Results From a Large, Statewide, Trauma Database. Urology. 2016;97:92-7. | Study population: adult patients (≥ 14 years) with polytrauma or trauma-related severe injury. |
| Hegele A, Lefering R, Hack J, Ruchholtz S, Hofmann R, Kuhne CA. [Operative interventions of urologic traumata in severe injured patients in the acute phase]. Urologe (Ausg A). 2016;55(4):506-13. | Intervention: surgical/interventional measures in the field of urology. |
| Ho P, Hellenthal NJ. Independent predictors of mortality for patients with traumatic renal injury. World Journal of Urology. 2021;05:05. | Study population: adult patients (≥ 14 years) with polytrauma or trauma-related severe injury. |
| Keihani S, Xu Y, Presson AP, Hotaling JM, Nirula R, Piotrowski J, et al. Contemporary management of high-grade renal trauma: Results from the American Association for the Surgery of Trauma Genitourinary Trauma study. J Trauma Acute Care Surg. 2018;84(3):418-25. | Intervention: surgical/interventional measures in the field of urology. |
| Laal M, Khodadadi F, Zarei MR. Renal trauma management in 8 cities of Iran. Acta Medica Iranica. 2009;47(1):61-4. | Study population: adult patients (≥ 14 years) with polytrauma or trauma-related severe injury. |
| McClung CD, Hotaling JM, Wang J, Wessells H, Voelzke BB. Contemporary trends in the immediate surgical management of renal trauma using a national database. The Journal of Trauma and Acute Care Surgery. 2013;75(4):602-6. | Study population: adult patients (≥ 14 years) with polytrauma or trauma-related severe injury. |
| McPhee M, Arumainayagam N, Clark M, Burfitt N, DasGupta R. Renal injury management in an urban trauma centre and implications for urological training. Ann R Coll Surg Engl. 2015;97(3):194-7. | Study type: comparative, prospective studies, comparative registry data, case-control studies (no non-comparative-studies and no retrospective cohort studies) & cross-sectional studies as well as Systematic Reviews* (based on the aforementioned primary stentypen), die relevante (klinische) Endpunkte berichten. |
| Narendra JB, Ratkal C, Keshavamurthy R, Karthikeyan V. Clinical profile of patients with renal trauma: A cross-sectional observational study. Urological Science. 2020;31(3):131-5. | Study population: adult patients (≥ 14 years) with polytrauma or trauma-related severe injury. |
| Schellenberg M, Benjamin E, Piccinini A, Inaba K, Demetriades D. Selective nonoperative management of renal gunshot wounds. The Journal of Trauma and Acute Care Surgery. 2019;87(6):1301-7. | Study population: adult patients (≥ 14 years) with polytrauma or trauma-related severe injury. |
| Torrance R, Kwok A, Mathews D, Elliot M, Baird A, Lucky MA. Management of renal injury in a UK major trauma centre. Trauma. 2020;22(1):26-31. | Study type: comparative, prospective studies, comparative registry data, case-control studies (no non-comparative-studies and no retrospective cohort studies) & cross-sectional studies as well as Systematic Reviews* (based on the aforementioned primary stentypen), die relevante (klinische) Endpunkte berichten. |
| Xu H, Min X, Li Y, Yang L, Ren Y. A Comparative Study of Conservation, Endovascular Embolization Therapy, and Surgery for Blunt Renal Trauma. Medical Science Monitor. 2020;26:e922802. | Study population: adult patients (≥ 14 years) with polytrauma or trauma-related severe injury. |
| Yeung LL, McDonald AA, Como JJ, Robinson B, Knight J, Person MA, et al. Management of blunt force bladder injuries: A practice management guideline from the Eastern Association for the Surgery of Trauma. The Journal of Trauma and Acute Care Surgery. 2019;86(2):326-36. | Study type: comparative, prospective studies, comparative registry data, case-control studies (no non-comparative-studies and no retrospective cohort studies) & cross-sectional studies as well as Systematic Reviews* (based on the aforementioned primary stentypen), die relevante (klinische) Endpunkte berichten. |

# S4 Evidence Table

##### Nephrectomy, organ-preserving surgical treatment

| Study: Reference, aim, design, setting | Participants: selection criteria, characteristics | N Participants; Intervention (IG) vs. Control group (CG) | Main outcomes | Assessment: LoE, risk of bias; Conclusions |
| --- | --- | --- | --- | --- |
| El Hechi (2020)  “Contemporary management of penetrating renal trauma - A national analysis.” Injury. 2020; 51(1): 32-8.  Study design  Comparative registry study  (ACS Trauma Quality and Improvement Program)  Aim of the study  In this analysis of a nationwide trauma database, our primary aim is to determine the rate and predictors of failure of NOM (f-NOM) for patients with PRI. Our secondary aim is to compare outcomes between patients managed operatively and nonoperatively.  Setting  USA, 2010-2016 | Inclusion criteria   - Patients aged 16 years and older with penetrating renal trauma   Exclusion criteria   - patients who mapped onto more than one AAST injury grade, had unknown ED dispositions, or died in the ED or operating room directly from the ED   IO vs NOM   - Age [y], median (IQR)   IG: 28.0 (22.0, 37.0) CG: 27.0 (22.0, 36.0), p=0.074  Male, n (%)  IG: 1344 (88.9) CG: 303 (91.8), p=0.12  ISS median (IQR)  IG: 25.0 (18.0, 34.0) CG:18.0 (14.0, 26.0), p<0.001  AIS Head >3 IG: 32 (2.1) CG: 13 (3.9), p=0.052  AIS Chest>3 IG: 709 (46.9) CG: 153 (46.4), p=0.86  Concomitant abdominal injuries, n (%) IC: 1315 (87.0) CG: 187 (56.7), p<0.001  Mechanism of Injury p<0.001  Gunshot Wounds  IG: 1344 (88.9) CG: 195 (59.1)  Stab Wounds IG: 168 (11.1) CG: 135 (40.9)  Comorbidity  Chronic Renal Failure  IG: 2 (0.1) CG: 2 (0.6), p=0.30  Diabetes Mellitus IG: 44 (2.9) CG: 5 (1.5), p=0.20  Hypertension  IG: 114 (7.5) CG: 18 (5.5), p=0.18 | Participants  N=1842 patients  Study groups  IG: immediate operation (IO) (N=1512)  CG1: NOM (N=330)  CG2: s-NOM (N=304)  CG3: f-NOM (N=26)  The IO group included patients who, in the first 4 h of their admission, underwent a nephrectomy, a renorraphy, or underwent abdominal exploration with no intervention on the injured kidney.  The NOM group included patients who did not undergo an abdominal exploration or an operative intervention on the kidney within the initial 4 h of admission Patients in the NOM group were either treated expectantly, received a ureteral stent, a nephrostomy, or angioembolization of the kidney In the NOM group, an abdominal exploration, a nephrectomy, or a renorraphy after 4 h of admission qualified as failure of NOM (f-NOM). NOM was considered successful (s-NOM) if abdominal exploration, nephrectomy, or renorraphy did not occur. | Severe Sepsis n (%)  IG:71 (4.7) CG1:1 (0.3), p<0.001  CG2:1 (0.3) CG3: 0 (0.0), p=1.00  IG: 71 (4.7) CG2:1 (0.3), p=n.r.  Length of stay [d] median (IQR)  IG: 14.0 (8.0, 25.0) CG1: 6.0 (4.0, 12.0), p<0.001  CG2: 6.0 (4.0, 10.0) CG3: 20.0 (11.0, 34.0) , p<0.001  IG: 14.0 (8.0, 25.0) CG2: 6.0 (4.0, 10.0), p=n.r.  Length of ICU stay [d] median (IQR)  IG: 5.0 (3.0, 13.0) CG1: 3.0 (2.0, 5.0), p<0.001  CG2: 3.0 (2.0, 4.0) CG3: 6.0 (3.0, 12.0), p<0.001  IG: 5.0 (3.0, 13.0) CG2: 3.0 (2.0, 4.0), p=n.r.  Ventilation [d] median (IQR)  IG: 3.0 (2.0, 8.0) CG1: 2.0 (2.0, 4.0), p=0.003  CG2: 2.0 (2.0, 4.0) CG3: 3.0 (2.0, 5.0), p=0.24  IG: 3.0 (2.0, 8.0) CG2: 2.0 (2.0, 4.0), p=n.r.  Inpatient Morbidity n (%)  IG: 482 (31.9) CG1: 37 (11.2), p<0.001  CG2: 27 (8.9) CG3: 10 (38.5), p<0.001  IG: 482 (31.9) CG2: 27 (8.9), p=n.r.  Need for Dialysis n (%)  IG: 42 (2.8) CG1: 0 (0.0), p<0.001  CG2: 0 (0.0) CG3: 0 (0.0), p=n.a.  IG: 42 (2.8)) CG2: 0 (0.0), p=n.r.  Acute Kidney Injury n (%)  IG: 144 (9.5) CG1: 7 (2.1), p<0.001  CG2: 5 (1.6) CG3: 2 (7.7), p=0.098  IG: 144 (9.5) CG2: 5 (1.6), p=n.r.  Urinary tract infection n (%)  IG: 58 (3,8) CG1: 3 (0,9), p=0,13  CG2: 6 (2,0) CG3: 1 (3,8), p=0,44  IG: 58 (3,8) CG2: 6 (2,0), p=n.r.  Ventilator associated pneumonia n (%)  IG: 108 (7,1) CG1: 6 (1,8), p<0,001  CG2: 4 (1,3) CG3: 2 (7,7), p=0,074  IG: 108 (7,1) CG2: 4 (1,3), p=n.r. | Level of evidence  2b  Risk of bias  Selection bias: –  Performance bias: –  Attrition bias: +  Detection bias: ?  Authors’ conclusion  “NOM is highly successful in selected patients. Concomitant abdominal injuries and higher grade AAST injuries predict NOM failure and should be considered when selecting patients for IO or NOM.”  Reviewers’ conclusion  There are different significant outcomes comparing IG vs. CG1 and CG2 vs. CG3.The results should be interpreted with caution due to missing information on detection bias. Furthermore, there is a risk of selection and performance bias. |
| +: low risk; –: high risk; ?: unclear risk; AAST: American Association for the Surgery of Trauma; AIS: Abbreviated Injury Scale; CG: control group; d: days; ED: Emergeny department; f-NOM: failure of NOM; ICU: intensive care unit; IG: intervention group; IO: immediate operation; IQR: Interquartile Range; ISS: injury severity score; LoE: level of evidence; m: months; n.a.: not applicable; NOM: nonoperative management; n.r.: not reported; s-NOM: successfull NOM; y: years | | | | |

##### Therapy of extraperitoneal urinary bladder ruptures

| Study: Reference, aim, design, setting | Participants: selection criteria, characteristics | N Participants; Intervention (IG) vs. Control group (CG) | Main outcomes | Assessment: LoE, risk of bias; Conclusions |
| --- | --- | --- | --- | --- |
| Anderson (2020)  “Current Management of Extraperitoneal Bladder Injuries: Results from the Multi-Institutional Genito-Urinary Trauma Study (MiGUTS).” Journal of Urology. 2020; 204(3): 538-44.  Study design  Prospective cohort study  Aim of the study  We studied the current management trends for extraperitoneal bladder injuries and evaluated the use of operative repair versus catheter drainage, and the associated complications with each approach.  Setting  USA, 2013-2018 | Inclusion criteria   - patients with extraperitoneal bladder   Exclusion criteria   - patients with an intraperitoneal bladder injury component and those who died within 24 hours of admission. - died within 24 hours of hospital arrival   Age [y], mean (SD)  IG: 44.7 (18.6) CG: 35.0 (14.2), p<0.001  Male, n (%)  IG: 54 (60) CG: 52 (78), p=0.02  ISS mean (SD)  IG: 28.4 (13.1)  CG: 23.0 (11.2), p=0.01  Concomitant injuries n (%)  Overall IC: 82 (91) CG: 57 (85), p=0.24  Pelvic fracture IG: 79 (89) CG: 45 (67), p<0.001  Bladder neck injury IG: 5 (6) CG: 14 (21), p=0.004  Urethral injury IG: 7 (8) CG: 8 (12), p=0.38  Colon injury  IG: 6 (7) CG: 10 (15), p=0.09  Rectal injury  IG: 4 (4) CG: 14 (21), p<0.001 | Participants  N=157 patients  Study groups  IG: catheter drainage (N=90)  CG: operative repair (N=67)  Patients were grouped as either catheter drainage or operative repair based upon their initial management within the first 4 days after hospital admission. | Length of stay [d], median (IQR)  IG: 12 (6-20) CG: 12 (6-21), p=0.81  Length of ICU stay [d], median (IQR)  IG: 5 (3-13) CG: 6 (2-15), p=0.97  Urological complications, n (%)  IG: 16 (18) CG: 11 (16), p=0.82  Orthopedic complications, n (%)  IG: 5 (6) CG: 5 (7), p=0.74  Significant complications were defined as the presence of urological or orthopedic conditions such as pelvic infection/urinoma, persistent urinary extravasation, urinary tract fistula nonunion fractures, hardware infection or removal, and pelvic osteomyelitis. | Level of evidence  2b  Risk of bias  Selection bias: –  Performance bias: –  Attrition bias: +  Detection bias: ?  Authors’ conclusion  “We found no significant difference in complications between the initial management strategies of catheter drainage and operative repair.”  Reviewers’ conclusion  There is no significant difference in complications between IG and CG. The results should be interpreted with caution due to missing information on detection bias. Furthermore, there is a risk of selection and performance bias. |
| +: low risk; –: high risk; ?: unclear risk; CG: control group; CI: Confidence Interval; d: days; ICU: intensive care unit; IG: intervention group; IQR: Interquartile Range; ISS: injury severity score; LoE: Level of evidence; m: months; n.r.: not reported; SD: standard deviation; y: years | | | | |
